# Supplementary figures and images for: CROP2, a Retriever–PROPPIN complex mediating protein export from endosomes to the plasma membrane in human cells
Source: eLife. 2026 Jul 24;14:RP109403. doi: 10.7554/eLife.109403 (PMC13399975; doi:10.7554/eLife.109403)

Figure 1- Source Data 1 Uncropped western blot for Figure 1 D

Figure 1 D

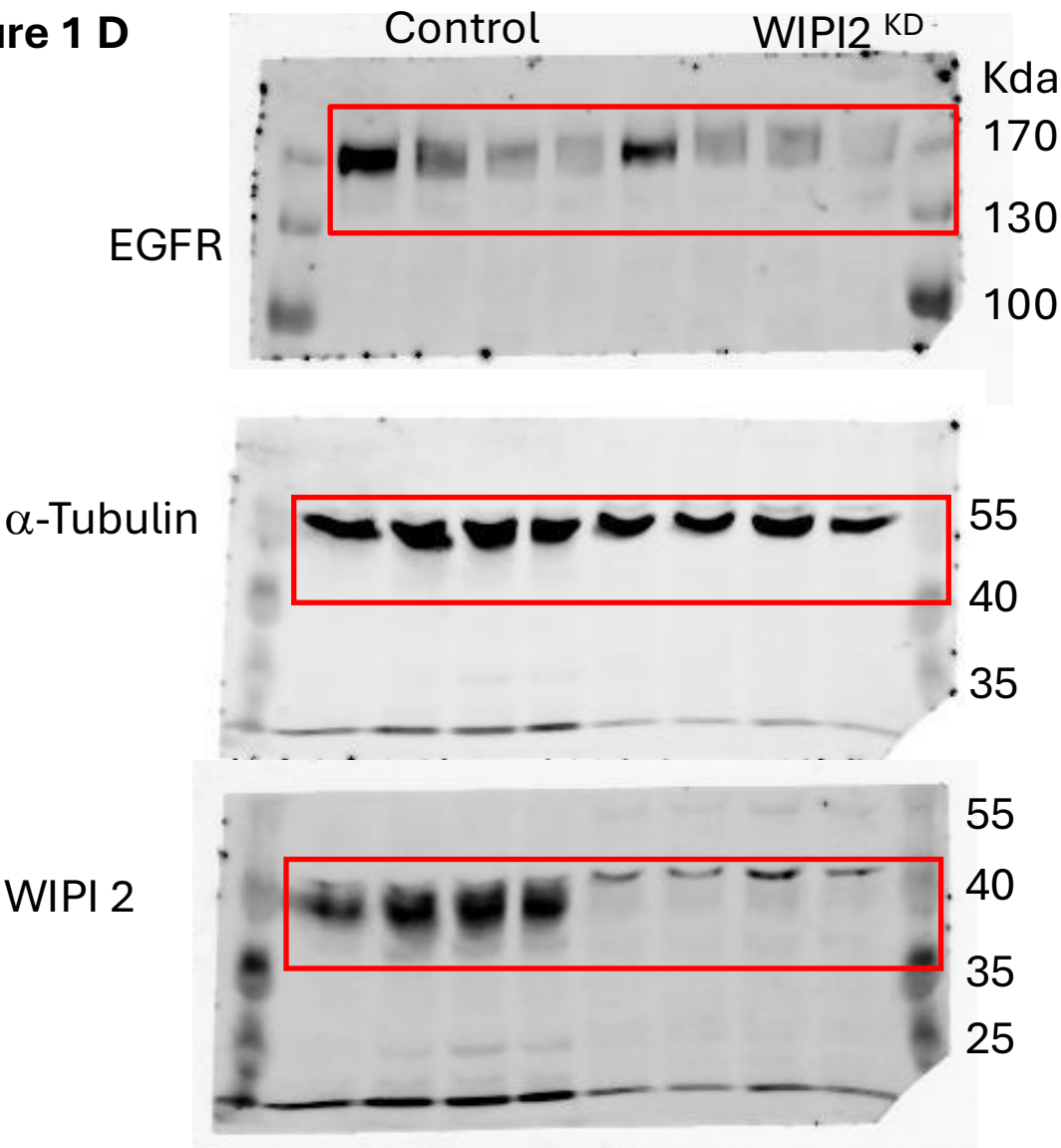

Supplement: Figure 1—source data 1. [file elife-109403-fig1-data1.zip › Figure 1-Source Data 1/Figure 1- Source Data 1.pdf]

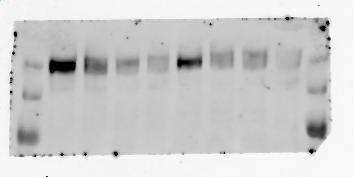

Supplement: Figure 1—source data 2. [file elife-109403-fig1-data2.zip › Figure 1-Source Data 2/egfr.jpg]

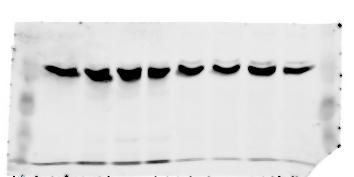

Supplement: Figure 1—source data 2. [file elife-109403-fig1-data2.zip › Figure 1-Source Data 2/tubulin.jpg]

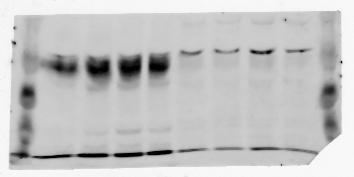

Supplement: Figure 1—source data 2. [file elife-109403-fig1-data2.zip › Figure 1-Source Data 2/wipi2.jpg]

Figure S1

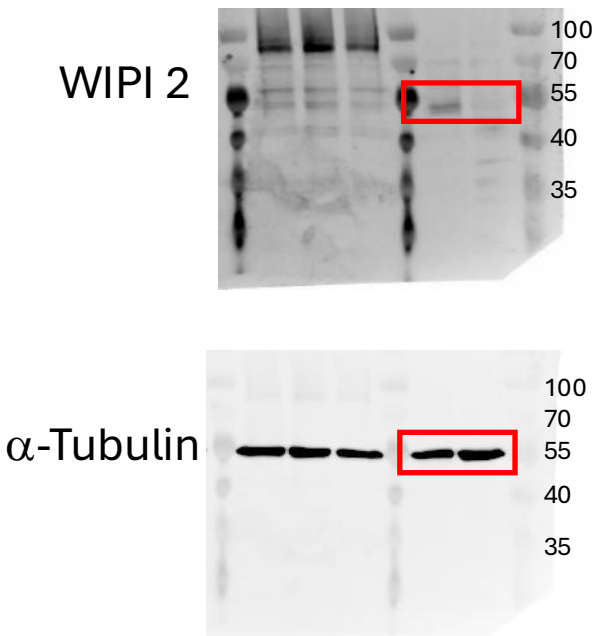

Supplement: Figure 1—figure supplement 1—source data 1. [file elife-109403-fig1-figsupp1-data1.zip › Figure S1-source data 1/Figure S1-source data 1.pdf]

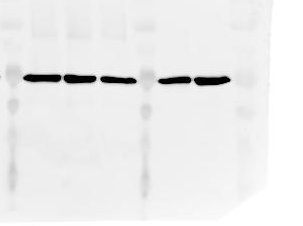

Supplement: Figure 1—figure supplement 1—source data 2. [file elife-109403-fig1-figsupp1-data2.zip › Figure S1-source data 2/tubulin.tif]

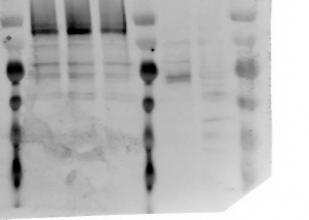

Supplement: Figure 1—figure supplement 1—source data 2. [file elife-109403-fig1-figsupp1-data2.zip › Figure S1-source data 2/wipi2.tif]

Figure S3- Source Data 1 Uncropped western blot for Figure S3

Figure S3 A

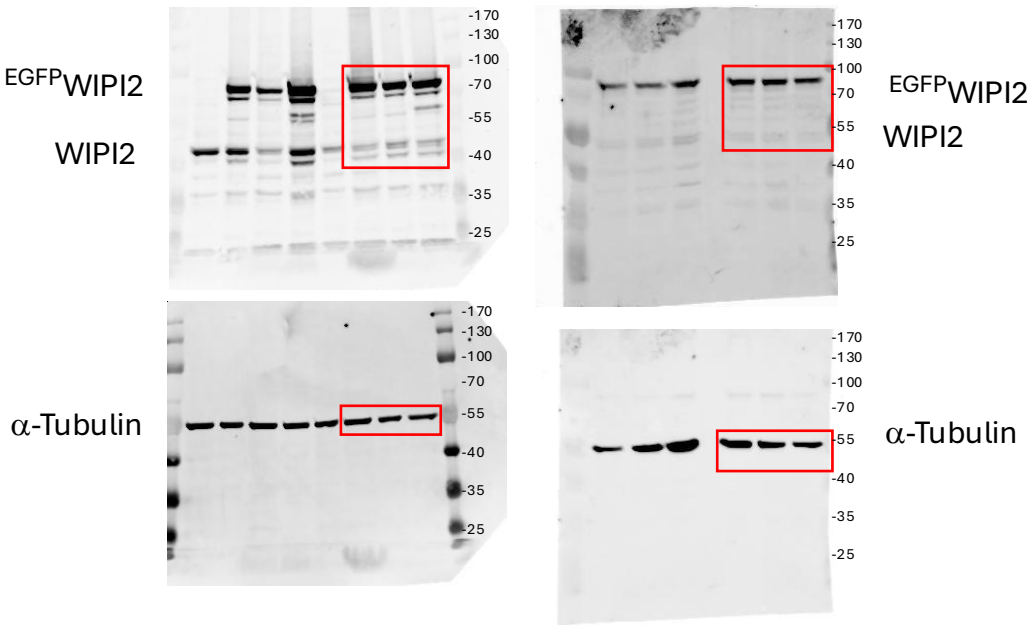

Figure S3 C

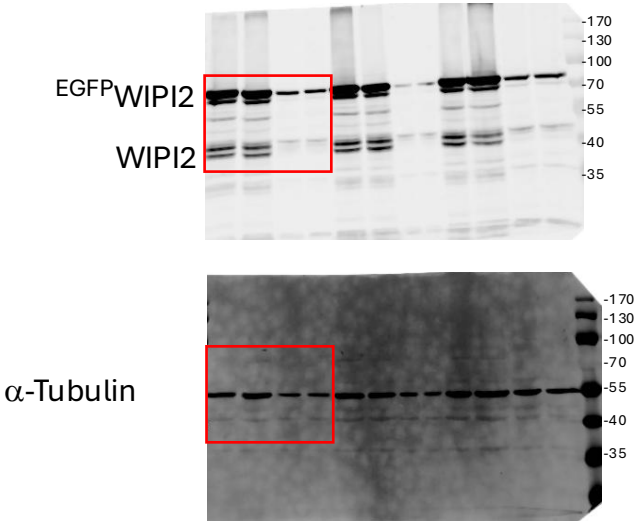

Supplement: Figure 4—figure supplement 1—source data 1. [file elife-109403-fig4-figsupp1-data1.zip › Figure S3-source data 1/Figure S3- Source Data 1 .pdf]

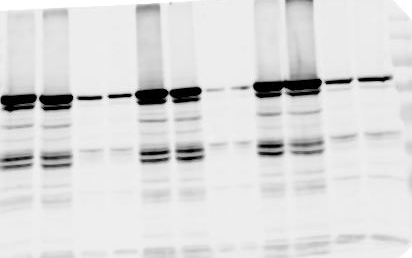

Supplement: Figure 4—figure supplement 1—source data 2. [file elife-109403-fig4-figsupp1-data2.zip › Figure S3-source data 2/w2wt sloop.tif]

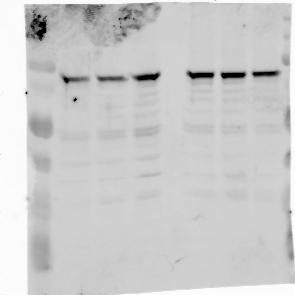

Supplement: Figure 4—figure supplement 1—source data 2. [file elife-109403-fig4-figsupp1-data2.zip › Figure S3-source data 2/w2variants level in wipi2KD.jpg]

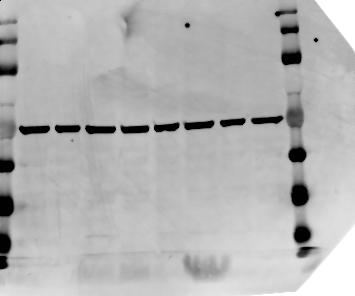

Supplement: Figure 4—figure supplement 1—source data 2. [file elife-109403-fig4-figsupp1-data2.zip › Figure S3-source data 2/tub in ctrl.jpg]

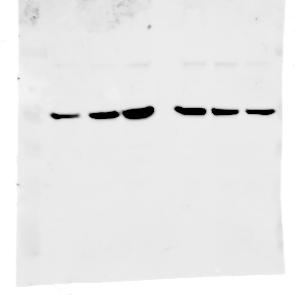

Supplement: Figure 4—figure supplement 1—source data 2. [file elife-109403-fig4-figsupp1-data2.zip › Figure S3-source data 2/tub in wipi2 kd.jpg]

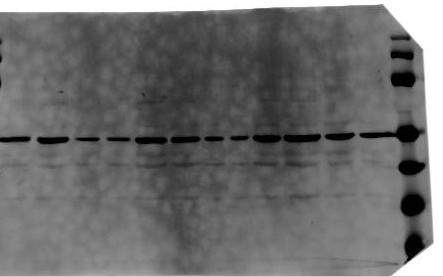

Supplement: Figure 4—figure supplement 1—source data 2. [file elife-109403-fig4-figsupp1-data2.zip › Figure S3-source data 2/tubulin wt sloop.tif]

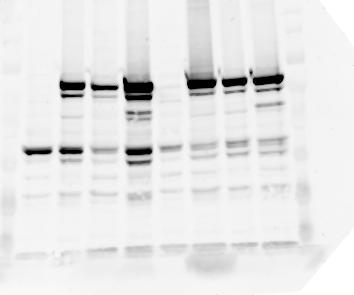

Supplement: Figure 4—figure supplement 1—source data 2. [file elife-109403-fig4-figsupp1-data2.zip › Figure S3-source data 2/wipi2 variants level in ctrl.jpg]

Figure 6- Source Data 1 Uncropped western blot for Figure 6

Figure 6 A

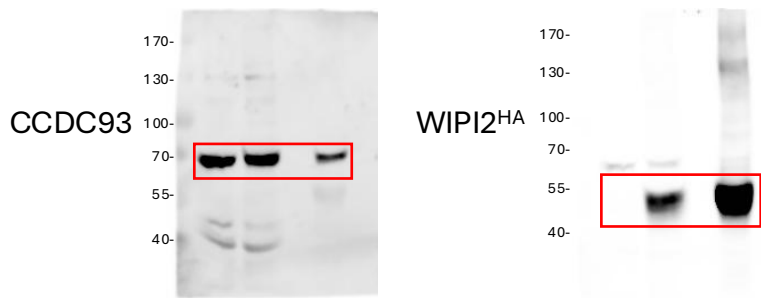

Figure 6 B

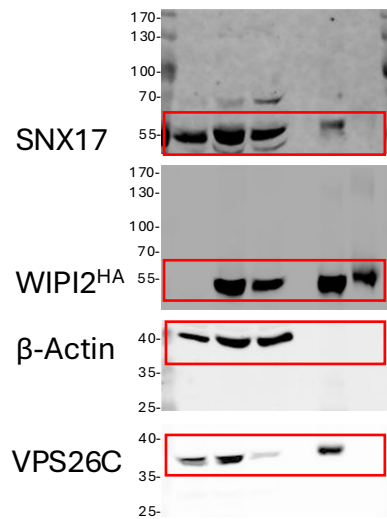

Figure 6 C

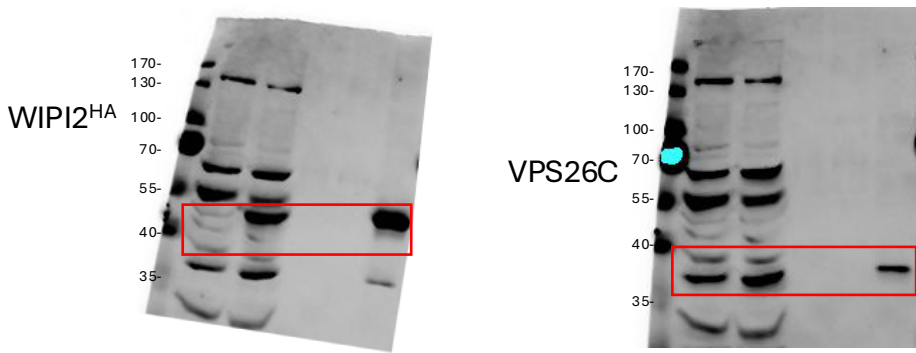

Figure 6 D

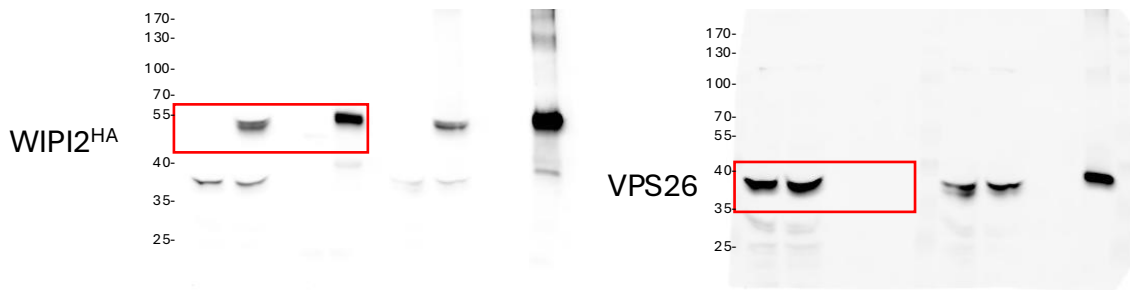

Supplement: Figure 6—source data 1. [file elife-109403-fig6-data1.zip › Figure 6-source data 1/Figure 6-source data 1.pdf]

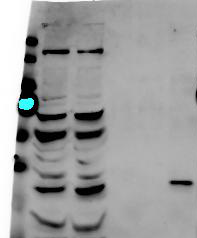

Supplement: Figure 6—source data 2. [file elife-109403-fig6-data2.zip › Figure 6-source data 2/VPS26C W2 .tif]

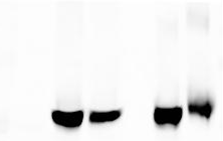

Supplement: Figure 6—source data 2. [file elife-109403-fig6-data2.zip › Figure 6-source data 2/ha w2 in vps26 c kd.tif]

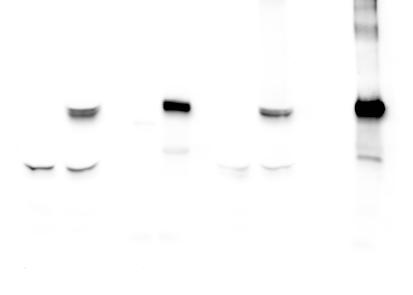

Supplement: Figure 6—source data 2. [file elife-109403-fig6-data2.zip › Figure 6-source data 2/ha gel A-H buono.jpg]

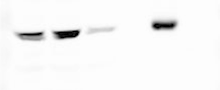

Supplement: Figure 6—source data 2. [file elife-109403-fig6-data2.zip › Figure 6-source data 2/vps26 c in kd.tif]

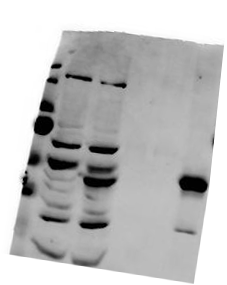

Supplement: Figure 6—source data 2. [file elife-109403-fig6-data2.zip › Figure 6-source data 2/HA WIPI2 vps26c.tif]

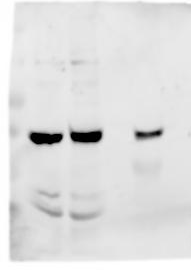

Supplement: Figure 6—source data 2. [file elife-109403-fig6-data2.zip › Figure 6-source data 2/ccdc93.tif]

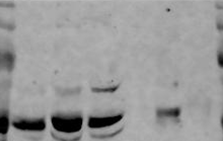

Supplement: Figure 6—source data 2. [file elife-109403-fig6-data2.zip › Figure 6-source data 2/snx17 inkd.tif]

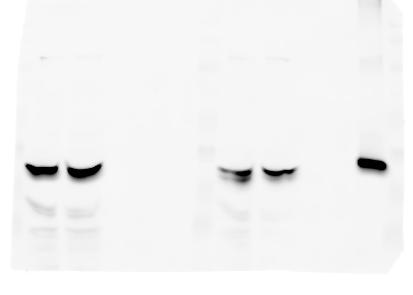

Supplement: Figure 6—source data 2. [file elife-109403-fig6-data2.zip › Figure 6-source data 2/vps26 gel A-H.jpg]

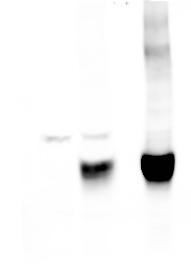

Supplement: Figure 6—source data 2. [file elife-109403-fig6-data2.zip › Figure 6-source data 2/w2 ha.tif]

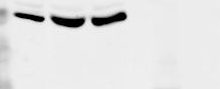

Supplement: Figure 6—source data 2. [file elife-109403-fig6-data2.zip › Figure 6-source data 2/actin in vps26c kd.tif]

Figure 7- Source Data 1 Uncropped western blot for Figure 7

Figure 7 A

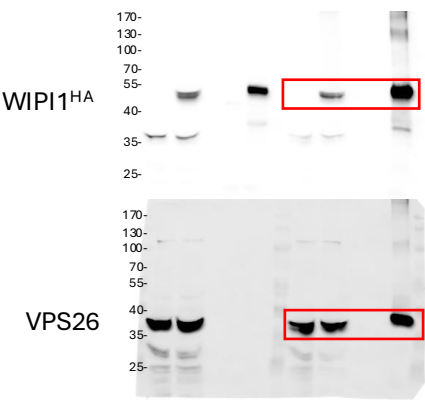

Figure 7 B

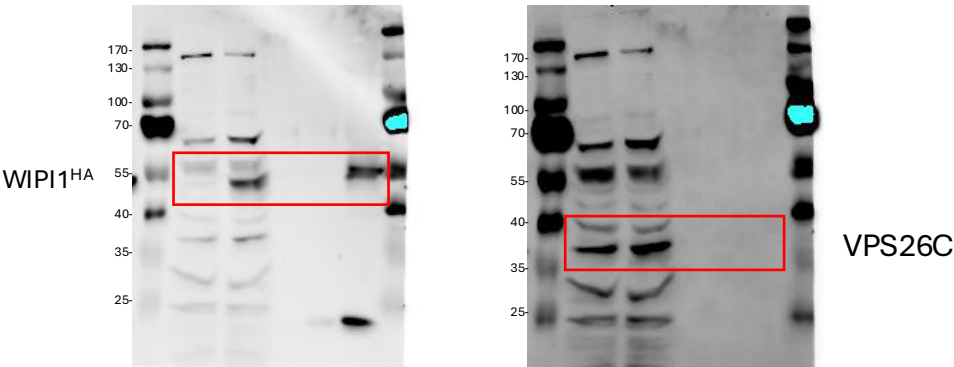

Figure 7 C

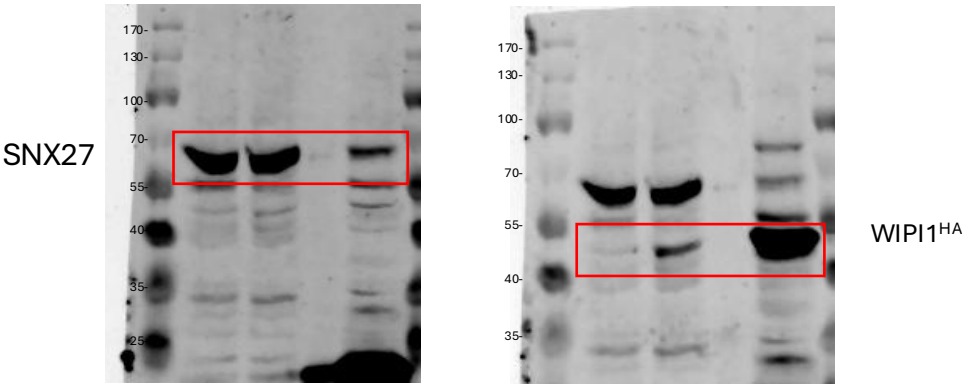

Supplement: Figure 7—source data 1. [file elife-109403-fig7-data1.zip › Figure 7-Source data 1/Figure 7-source data 1.pdf]

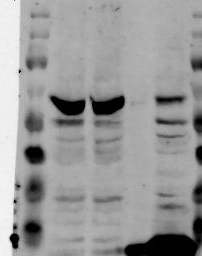

Supplement: Figure 7—source data 2. [file elife-109403-fig7-data2.zip › Figure 7-source data 2/SNX27 W1.tif]

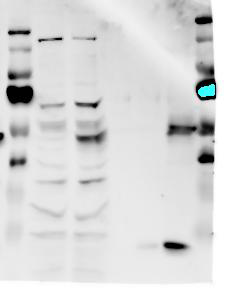

Supplement: Figure 7—source data 2. [file elife-109403-fig7-data2.zip › Figure 7-source data 2/HA WIPI1 VPS26C .tif]

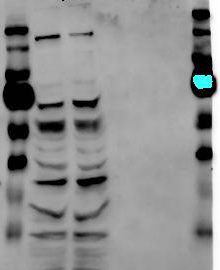

Supplement: Figure 7—source data 2. [file elife-109403-fig7-data2.zip › Figure 7-source data 2/VPS26C W1.tif]

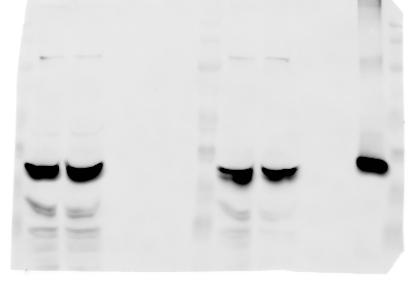

Supplement: Figure 7—source data 2. [file elife-109403-fig7-data2.zip › Figure 7-source data 2/vps26 gel A-H more exp.jpg]

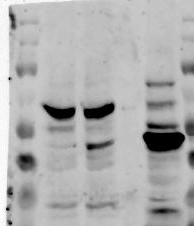

Supplement: Figure 7—source data 2. [file elife-109403-fig7-data2.zip › Figure 7-source data 2/HA W1 SNX27.tif]

Figure 8- Source Data 1 Uncropped western blot for Figure 8

Figure 8 A

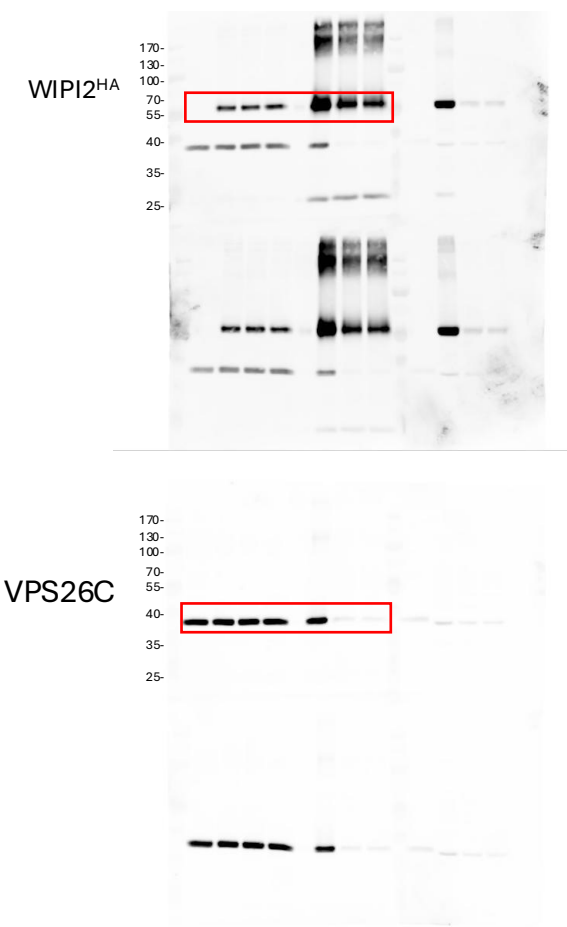

Supplement: Figure 8—source data 1. [file elife-109403-fig8-data1.zip › Figure 8-source data 1/Figure 8-source data 1.pdf]

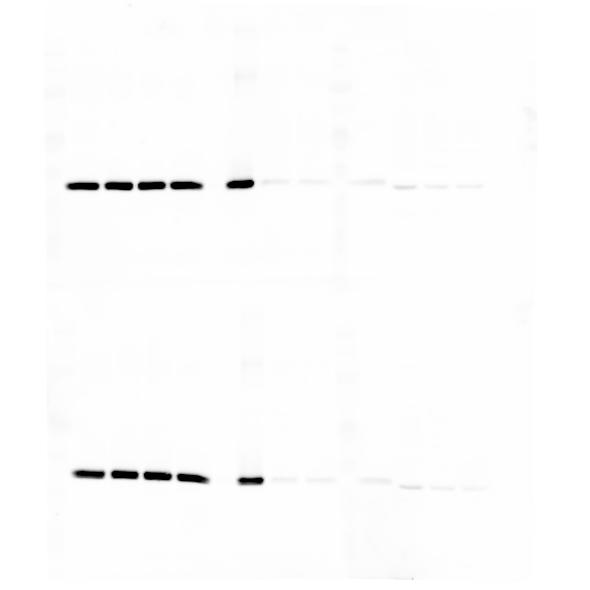

Supplement: Figure 8—source data 2. [file elife-109403-fig8-data2.zip › Figure 8-source data 2/vps26C .jpg]

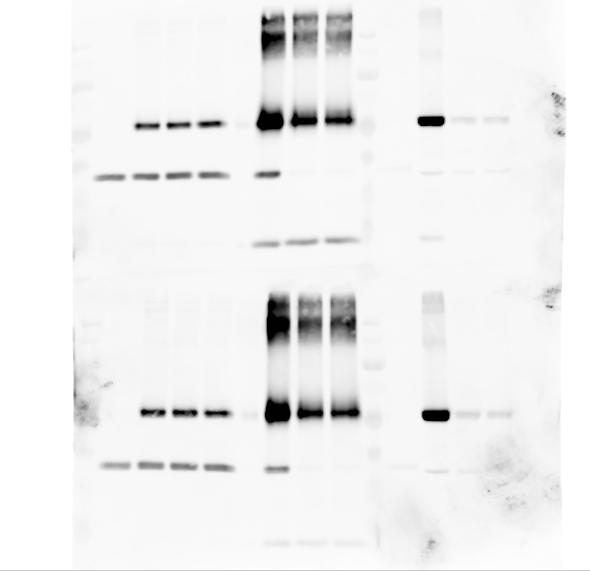

Supplement: Figure 8—source data 2. [file elife-109403-fig8-data2.zip › Figure 8-source data 2/wipi2 ha.jpg]
