# Supplementary material for: CROP2, a Retriever–PROPPIN complex mediating protein export from endosomes to the plasma membrane in human cells
Source: eLife. 2026 Jul 24;14:RP109403. doi: 10.7554/eLife.109403 (PMC13399975; doi:10.7554/eLife.109403)
Supplement: Supplementary file 1. [file elife-109403-supp1.docx]

**Appendix 1:** Sequences of WIPI1 and WIPI2 that had been used for mutagenesis and for generating tagged alleles.

**WIPI2-Gly-Gly-Gly-Ser- Gly-Gly-Gly-Ser-HA-HA-Gly-HA-HA-STOP**

ATGAACCTGGCGAGCCAGAGCGGGGAGGCCGGCGCCGGCCAGCTGCTCTTCGCCAACTTCAACCAGGACAACACGTCCCTAGCTGTTGGTAGTAAGTCCGGTTATAAATTTTTCTCCCTTTCTTCTGTGGATAAGCTGGAACAGATCTATGAATGCACCGATACGGAAGATGTGTGCATTGTAGAGAGATTGTTCTCCAGCAGCCTAGTGGCCATCGTCAGCCTTAAAGCACCAAGGAAGCTAAAGGTTTGCCACTTTAAGAAGGGAACTGAGATCTGCAACTACAGCTACTCCAACACGATTCTGGCTGTGAAGCTCAACAGGCAGAGGCTGATAGTATGCCTGGAGGAGTCCCTGTACATCCACAACATTCGGGACATGAAGGTGCTGCATACGATCAGGGAGACGCCTCCAAACCCTGCAGGCCTGTGTGCGCTGTCAATCAACAACGACAACTGCTACTTGGCGTACCCAGGGAGCGCGACCATCGGAGAGGTGCAGGTCTTCGATACCATTAATTTGAGAGCTGCAAACATGATTCCGGCTCACGACAGTCCTTTAGCGGCACTGGCCTTTGACGCAAGTGGAACTAAACTTGCCACGGCTTCGGAGAAGGGGACCGTGATTAGGGTATTTTCCATTCCAGAAGGACAAAAACTCTTTGAGTTTCGGAGAGGAGTAAAGAGGTGCGTGAGCATCTGCTCCCTGGCCTTCAGCATGGACGGCATGTTCCTCTCCGCCTCCAGCAACACTGAGACCGTGCACATCTTCAAACTCGAGACTGTGAAAGAAAAACCCCCAGAGGAGCCCACCACCTGGACCGGGTACTTCGGGAAAGTGCTCATGGCCTCCACCAGCTACCTGCCTTCCCAAGTGACAGAAATGTTCAACCAGGGCAGAGCCTTCGCCACGGTCCGCCTGCCATTCTGCGGCCACAAAAACATCTGCTCGCTAGCCACAATTCAGAAGATCCCGCGGTTGTTGGTGGGTGCCGCCGACGGGTACCTGTACATGTACAACCTGGACCCCCAGGAGGGCGGCGAGTGTGCCCTGATGAAGCAGCACCGGCTGGACGGCAGTCTGGAAACGACCAATGAGATCTTGGACTCTGCCTCTCACGACTGCCCCTTAGTCACTCAGACATACGGCGCAGCTGCAGGAAAAGGTACTTACGTGCCTTCATCCCCAACGAGACTTGCCTACACAGACGACCTGGGTGCTGTGGGTGGCGCCTGCCTGGAGGACGAGGCCAGCGCCCTGCGCCTGGATGAGGACAGCGAGCACCCGCCCATGATTCTTCGGACTGACGGGGGCGGATCCGGCGGAGGCTCCTACCCATACGATGTGCCTGACTATGCCGGCTATCCCTATGACGTCCCTGACTATGCA**TGA**

**WIPI1-Gly-Gly-Gly-Ser- Gly-Gly-Gly-Ser-HA-HA-Gly-HA-HA-STOP**

ATGGAGGCCGAGGCCGCGGACGCTCCCCCGGGCGGGGTTGAGTCGGCGCTCAGCTGCTTCTCTTTCAACCAGGACTGCACATCCCTAGCAATTGGAACTAAAGCCGGGTATAAGCTGTTTTCTCTGAGTTCTGTGGAGCAGCTGGATCAAGTCCACGGAAGCAATGAAATCCCGGACGTCTACATCGTGGAGCGCCTCTTCTCCAGCAGCCTGGTGGTGGTAGTCAGTCACACAAAACCACGGCAGATGAACGTGTATCACTTCAAGAAAGGCACAGAGATCTGTAATTACAGCTACTCCAGCAACATCTTGTCCATAAGGCTGAACCGGCAAAGGCTGCTGGTTTGCCTAGAAGAGTCCATTTATATTCACAACATTAAAGACATGAAGCTGTTGAAGACCCTCCTGGATATTCCTGCAAACCCAACAGGTCTATGTGCTCTCTCTATCAACCATTCCAATTCTTACCTGGCCTATCCTGGAAGCCTGACTTCAGGGGAGATTGTGCTTTATGATGGAAACTCCCTGAAAACAGTCTGCACTATTGCTGCCCATGAGGGAACACTAGCTGCCATCACCTTCAATGCCTCAGGCTCCAAACTAGCAAGTGCGTCTGAAAAAGGCACAGTCATCCGGGTGTTCTCTGTCCCTGATGGGCAAAAGCTCTATGAGTTCCGGAGAGGGATGAAAAGGTATGTGACAATCAGCTCTCTAGTGTTCAGTATGGATTCACAATTCCTCTGCGCCTCCAGTAACACCGAGACGGTACACATCTTCAAGCTGGAACAGGTCACCAACAGTCGACCAGAAGAGCCTTCGACCTGGAGTGGCTACATGGGAAAGATGTTTATGGCTGCTACCAACTACCTCCCTACCCAGGTGTCAGACATGATGCATCAGGACAGGGCTTTTGCCACTGCACGCTTGAACTTCTCCGGACAGAGGAACATCTGTACCCTCTCAACGATCCAGAAGTTGCCACGGCTGCTAGTTGCGTCATCCAGTGGACACCTTTATATGTACAATTTGGATCCTCAGGATGGAGGAGAGTGTGTCTTAATCAAAACCCACAGCTTGCTTGGCTCAGGAACAACAGAAGAGAATAAAGAAAATGACCTCAGACCTTCCTTACCTCAGTCTTATGCAGCGACCGTAGCCAGACCAAGTGCATCTTCAGCCTCCACGGTGCCAGGTTATTCTGAGGACGGCGGGGCGCTGCGAGGAGAAGTTATTCCTGAACATGAGTTTGCGACGGGACCAGTGTGTCTTGATGATGAGAATGAGTTTCCTCCTATAATCTTGTGCCGTGGAAATCAGAAGGGCAAAACGAAGCAGTCAGGGGGCGGATCCGGCGGAGGCTCCTACCCATACGATGTGCCTGACTATGCCGGCTATCCCTATGACGTCCCTGACTATGCA**TGA**
