# Supplementary material for: CROP2, a Retriever–PROPPIN complex mediating protein export from endosomes to the plasma membrane in human cells
Source: eLife. 2026 Jul 24;14:RP109403. doi: 10.7554/eLife.109403 (PMC13399975; doi:10.7554/eLife.109403)
Supplement: Peer review history

# Editor report: eLife Assessment

**DOI:** 10.7554/eLife.109403.3.sa0

**Authors:** Francesca Bottanelli

**Affiliations:** Freie Universität Berlin Germany

**Keywords:** Compelling, Important

The authors present **important** evidence for a WIPI2-Retriever complex (termed CROP2) that couples cargo selection to carrier fission at endosomes. CROP2 appears to function analogously to the previously described CROP1 complex, formed by WIPI1 and Retromer, with which it shares structural similarities. They provide **compelling** evidence that CROP1 and CROP2 regulate the trafficking of distinct subsets of cargoes; however, the cellular evidence for the existence of these distinct complexes is mostly inferred from immunoprecipitation analysis and would benefit from further validation.

# Referee report: Reviewer #1 (Public review)

**DOI:** 10.7554/eLife.109403.3.sa1

WIPI1 is a PROPPIN family protein that has been implicated in Retromer-mediated membrane fission events. Although the cargos that it has been tested to be important for are diverse, one of the cargos that is unaffected is Beta1-Integrin. This leads the authors to assess another PROPPIN family protein - WIPI2, which is a homolog of WIPI1. KD using siRNA is effective and had no consequences on LAMP1, EGFR trafficking or GLUT1 trafficking. Integrin-B1, however, had a large and significant defect in its recycling from the endosome, with a clear endosomal colocalisation. Complementation experiments with WT WIPI2 recovered the phenotype, but various mutant WIPI2 complements resulted in elongated tubules, and there was also a dominant negative effect of the mutant. Integrin is a classic retriever cargo, so the authors rationalise that WIPI2 may be playing a role with retriever that WIPI1 plays with retromer. To assess this, they perform a set of immunoprecipitations. SNX17, the retriever-associated sorting nexin, co-IPs with WIPI2 in a VPS26C-dependent manner. VPS26C but not VPS26 co-IPs with WIPI2, and the reciprocal with WIPI1. These interactions were not present for the FSSS mutation of WIPI2. WIPI2 localises to Rab11 endosomes mainly, as does retriever. Mutations of WIPI2 not only affected WIPI2 localisation, but also VPS35L mutations, indicating that there is a functional relationship between the two.

Comments on revised version.

The reviewers have responded appropriately to all the points. I have no remaining concerns.

# Referee report: Reviewer #3 (Public review)

**DOI:** 10.7554/eLife.109403.3.sa2

Summary:

The manuscript of Mayer and colleagues analyzes the function of WIPI proteins in mammalian cells. The authors identified previously CROP as a complex consisting of WIPI1 and the retromer complex, primarily in yeast cells. In mammalian cells, both WIPI1 and WIPI2 exist, whereas retromer has a homologous complex termed retriever. The now find that WIPI2 can form a complex with retriever subunits. They name this complex CROP2. Their data further indicate that CROP2 and CROP1 have distinct substrate specificities as knock down of CROP2 subunits affect beta1 integrin sorting, whereas knock down of CROP1 affects EGFR and GLUT1. The further identify a similar sequence (FSSS) in both WIPI1 and WIPI2, which is required for their specific binding to retromer and retriever.

Strengths:

CROP1 and CROP2 seem to use similar features for their formation, and have different substrates, which is convincingly shown.

Weaknesses:

The analysis lacks information that this is a complex as claimed. It can be deduced from the immunoprecipitation analysis.

Comments on revised version.

The authors answered my questions and adjusted the text accordingly. Figure 10 was not part of the submitted version. It should be checked by the editor.

# Author comment: Author response

**DOI:** 10.7554/eLife.109403.3.sa3

**Authors:** Maria Giovanna De Leo, Andreas Mayer

**Affiliations:** University of Lausanne Epalinges Switzerland; University of Lausanne Epalinges Switzerland

The following is the authors’ response to the original reviews.

**Public Reviews:**

**Reviewer #1 (Public review):**

WIPI1 is a PROPPIN family protein that has been implicated in Retromer-mediated membrane fission events. Although the cargos that it has been tested to be important for are diverse, one of the cargos that is unaffected is Beta1-Integrin. This leads the authors to assess another PROPPIN family protein - WIPI2, which is a homolog of WIPI1. KD using siRNA is effective and had no consequences on LAMP1, EGFR trafficking or GLUT1 trafficking. Integrin-B1, however, had a large and significant defect in its recycling from the endosome, with a clear endosomal colocalisation. Complementation experiments with WT WIPI2 recovered the phenotype, but various mutant WIPI2 complements resulted in elongated tubules, and there was also a dominant negative effect of the mutant. Integrin is a classic retreiver cargo, so the authors rationalise that WIPI2 may be playing a role with retreiver that WIPI1 plays with retromer. To assess this, they perform a set of immunoprecipitations. SNX17, the retreiver-associated sorting nexin, co-IPs with WIPI2 in a VPS26C-dependent manner. VPS26C but not VPS26 co-IPs with WIPI2, and the reciprocal with WIPI1. These interactions were not present for the FSSS mutation of WIPI2. WIPI2 localises to Rab11 endosomes mainly, as does retriever. Mutations of WIPI2 not only affected WIPI2 localisation, but also VPS35L mutations, indicating that there is a functional relationship between the two.

On the whole, I find the manuscript compelling. The manuscript is very clearly written, the results are convincing and well performed. The flow of experiments is logical, and although not comprehensive in the subsequent mechanistic understanding, the fundamental findings are important and convincing. My comments below are, on the whole, minor and are intended to support the communication of the findings to the field.

We are happy that the reviewer has received our work quite positively.

(1) The IP interaction data were convincing; however, for me and some others, an interaction is only convincing when performed in vitro, and understood at a structural level. I do not suggest the authors do that in this case; however, I think, at a minimum, some sensible moderation of claims would be useful here.

Indeed, quantitative *in vitro* data on the affinities would be a nice addition. However, we have significant trouble to recombinantly express and purify well-behaved WIPI2 in sufficient quantities for such studies. We keep working in this direction but are not there yet.

We have now inserted a phrase into the discussion section highlighting this limitation: "Our immunoprecipitation assays cannot distinguish and more detailed structural and interaction studies with pure compounds will be necessary to elucidate the nature of this interaction". We nevertheless think that the the isoform specificity of the IPs, the effect of the point mutations in WIPI2 on these interactions, and the functional effects *in vivo* lend signficant support to the notion of a complex even if there is no proof of direct binding of WIPI2 to Retriever.

(2) I found the final localisation data and its interpretation confusing. My interpretation of that data would not be that the retreiver is relocalised, but rather that there is less of both recruited to the membrane and the remaining localisation distribution is shifted. In addition, I am not quite sure of the model here - is the idea that WIPI2 recruits retreiver, if that is the case, I find it hard to resolve with its role as a mediator of fission. Clarity would be appreciated here.

We are not quite sure what "final" localisation data the reviewer refers to, but we guess it is Fig. 9. This figure primarily provides *in vivo* evidence supporting the connection between Retriever and WIPI2. It does this by showing that the S67 substitution shifts both proteins. In WIPI2 wildtype cells, WIPI2 and VPS35L strongly colocalize in Rab11 compartments. S67 substitutions in WIPI2 abolish this localisation; WIPI2 shifts mainly to Rab5 compartments, where VPS35L shows only a moderate increase, and to Rab7 compartments, where VPS35L shows no increase at all.

We do not understand the reviewer's interpretation that less Retriever would be recruited to the membranes in the S67 variants. VPS35L remains completely associated with punctate, presumably membrane-bounded structures also in the mutants, providing no evidence for a detachment from the membrane. The same is observed in a WIPI2 knockdown. Therefore, we did not claim that WIPI2 is the main factor recruiting Retriever to the membrane, for which our experiments yield no hints. This does not exclude that the interaction of WIPI2 could strengthen membrane recruitment, or that two pools of Retriever exist, one interacting with Snx17 and another interacting with WIPI2, and that both link to each other in a coat. We did not dwell on this in the discussion because our experiments cannot distinguish these possibilities and were not conceived to analyse membrane recruitment of Retriever.

(3) I am concerned that the repeats being compared for statistical analysis are not biological repeats but technical repeats (cells in the same experiment). I should think the idea of the statistical comparison is to show experimental reproducibility and variability across biological repeats. Therefore, I would expect an appropriate number of biological repeats (3 or more minimum), to be the data compared in the statistical analysis and graphs. I think it is appropriate to average the technical repeats from each biological repeat. I find these to be useful resources https://doi.org/10.1083/jcb.202401074, https://doi.org/10.1083/jcb.200611141

The repeats being compared are biological repeats from independent experiments. This is described in Methods, where the reviewer may not have seen it. In order to make the independent experiments more evident in the figures, we have now colour coded the individual cell measurements from the three independent experiments. This allows to visualize both the individual data points, the average from each experiment and the variability across the independent experiments.

**Reviewer #2 (Public review):**

Summary:

The manuscript from De Leo and Mayer presents evidence that the PROPPIN protein, WIPI2, associates with the Retriever complex, and is required for the proper transport of the SNX17-Retriever cargo, beta1-integrin. This finding fits with prior papers from the Mayer lab, which showed that a related PROPPIN, WIPI1, is required for the transport of some SNX27-Retromer cargo, including GLUT1. The retromer and retriever complexes are architecturally similar. Importantly, they act at the same endosomes, and each transports cargo from endosomes to the plasma membrane. Thus, the possibility that each also requires a structurally related PROPPIN is of interest. However, the manuscript is incomplete, and the main claims are only partially supported.

Strengths:

The topic that PROPPIN proteins are important for the function of the Retromer and Retriever complexes expands our view of the trafficking complex.

Weaknesses:

Many important controls are missing. Several points that are made in the manuscript are only supported through a single approach.

We made a serious effort and implemented many suggestions of this reviewer, but orthogonal approaches are not always available or accessible.

**Reviewer #3 (Public review):**

Summary:

The manuscript of Mayer and colleagues analyzes the function of WIPI proteins in mammalian cells. The authors previously identified CROP as a complex consisting of WIPI1 and the retromer complex, primarily in yeast cells. In mammalian cells, both WIPI1 and WIPI2 exist, whereas retromer has a homologous complex termed retriever. They now find that WIPI2 can form a complex with retriever subunits. They named this complex CROP2. Their data further indicate that CROP2 and CROP1 have distinct substrate specificities as knockdown of CROP2 subunits affects beta1 integrin sorting, whereas knockdown of CROP1 affects EGFR and GLUT1. They further identify a similar sequence (FSSS) in both WIPI1 and WIPI2, which is required for their specific binding to retromer and retriever.

Strengths:

CROP1 and CROP2 seem to use similar features for their formation, and have different substrates, which is convincingly shown.

Weaknesses:

The analysis lacks information that this is a complex as claimed. It can be deduced from the interaction analysis, but was not shown.

It is of course desirable to obtain a detailed structural and *in vitro* characterisation of this interaction, which we have not provided because we currently do not have sufficient amounts of well-behaved source material for this. We nevertheless think that the interaction we show, which is strictly isoform-specific and dependent on single amino acid substitutions in a motif that in CROP1 is necessary for the interaction its recombinant subunits, supports that CROP2 is a similar a complex. We don't show a direct interaction but also don't claim in the manuscript that the interaction between WIPI2 and Retriever is direct and independent of additional factors.

**Recommendations for the authors:**

**Reviewing Editor Comments:**

As you will see, the reviewers generally value the contribution to the field, but they feel that some claims require additional experimental support.

(1) I have summarized the major points below.

(a) Both reviewers 1 and 2 agree that the quality of localization data presented in Figure 9 and S5-S7, and the interpretation of the data, could be improved. See comment 2 from reviewer 1 and comments 23, 24 and 25 from reviewer 2. They not only suggest ways to improve the presentation of the data, but additionally suggest improving the staining of the Rab11 marker and additionally explain the lack of co-localization between VPS35 and Rab5, which has been reported in the literature.

This impression was due to the fact that some figures showed projections of image stacks, which was not indicated clearly in the figure legend. We have changed this and now show single image planes throughout all figures.

(b) Both reviewers 1 and 3 note that the evidence supporting a functional WIPI2-Retriever complex *in vivo* is currently weak. We agree that additional biochemical data demonstrating the presence of the CROP1 and CROP2 complexes *in vivo* would strengthen the central message of the paper and elevate it to a more fundamental discovery.

We understood that the reviewers did not ask for further *in vivo* evidence but would welcome structural characterisation of the complex and quantitative binding data *in vitro* with purified proteins. Structural characterisation is out of scope of our study and *in vitro* binding studies have remained hampered by the fact that WIPI2 is hard to express and purify and not well behaved *in vitro*.

(c) All reviewers agree that the authors should carefully repeat their statistical analysis to account for the number of biological replicates. Reviewer 1 suggests publications that the authors could refer to.

The reviewers have probably overlooked the respective description in the methods section, where it had been stated that we analysed biological replicates from independent experiments. In graphs showing measurements from individual cells we now make this evident through colour coded dots, in which each colour represents data points stemming from an independent experiment. This makes it evident that the variance from experiment to experiment is low. The means (*n* = 3) were generally compared using a two-tailed unpaired t-test.

(d) Reviewer 2 additionally has various minor points that would greatly improve the readability and presentation of the work, and we recommend addressing (comments 1, 2, 3, 4, 12, 15, 17, 20, 27, 28, 29). All reviewers, in general, provide great minor suggestions. It would be great if the CROP1 and 2 complexes could be clearly introduced in each figure. We also agree that the WIPI2 CT labelling is confused and should be changed to "control" or similar.

Many of the points raised by this reviewer were actually quite minor or questions of personal preference, not major problems as stated in the review. Nevertheless, we found a number of useful suggestions in this review and have addressed these points as detailed in the response to reviewer 2.

(2) In addition to the major shared concerns laid out in the points above, reviewer 2 has some further minor suggestions:

(a) Comment 6. Could the author explain the discrepancies between the example blot shown in Figure 1D and the quantification (1E).

The two have actually been quite consistent. The reviewer might have mistaken the marker lane as the 0 min reference value to arrive at this impression. We have now removed the marker lane to avoid this.

(b) Comment 9 - could the authors clarify how surface labelling experiments were carried out?

This had been clearly described in the methods section, where this reviewer has probably not seen it.

(c) Comment 11 - The reviewer suggests normalizing the surface levels of markers to the cell area and not per cell. This is a reasonable suggestion.

The analysis had already been performed as proposed. This had been clearly described in the methods section, which the reviewer may not have looked at.

(d) Comment 19 "In Figure S4, the authors observe tubular structures. The authors should perform immunofluorescence with endosomal markers such as EEA1, LAMP1 and Retromer to determine the nature of the tubulovesicular structures." The authors could try a Rab4 or Rab11 overexpression plasmid to show whether these are elongated recycling tubules.

This has now been added.

**Reviewer #1 (Recommendations for the authors):**

Minor comments:

(1) The figures are not colourblind friendly, and should be changed to be so. Additionally, single colour images should be grayscale.

That was a good learning opportunity. We adapted the colour schemes of the images to make them more colourblind friendly, now using magenta, green, and white for the overlaps. In doing so we have relied on published recommendations, but we have not found a colourblind colleague to check the efficacy of this change.

(2) WIPI2^CT labels are confusing, as people may think they are a mutant. I suggest changing to "control" or similar.

These have been changed.

(3) "The effect was comparable to that of a knockdown of SNX17 (Figure 3 A, B)." On page 6. Based on this sentence, I was expecting to see a comparison to SNX17 KD, but it was not there as far as I can tell.

This statement referred to a publication by P.Cullen and collaborators. We have changed the wording and inserted the (missing) reference to make this clear.

**Reviewer #2 (Recommendations for the authors):**

The manuscript is modest. In addition, many of the claims should be better supported by the addition of orthogonal data. Moreover, the quality of some of the data presented needs to be improved. Overall, the manuscript requires better descriptions of the methods. In many figures, it was not clear how the experiments were performed.

The experimental descriptions that the reviewer refers to had been provided in the Methods section, where this reviewer may have overlooked them.

The paper should also be better organized. Some less important findings are in the main figures, whereas some critical results are in the supplemental figures. In addition, there were multiple issues with the readability of the paper, and the authors should consider using a professional editor to make the paper easier to read.

We had given the paper to colleagues who found it clear, and also Reviewer 1 has underlined its clarity. Nevertheless, we have re-phrased the manuscript in some parts to optimise it.

One of the main claims in the paper is that the FSSS motif of WIPI2, as well as a conserved amphipathic helix, is critical for WIPI2 function in the CROP2 complex. It is notable that these are the same regions that are also critical for the role of WIPI2 in autophagy (Gubas et al., 2024 PMID: 39152217). The authors should include this information in the manuscript and cite the paper.

Indeed. We mention this now in the introduction of the revised version.

Additional Major Issues:

While some of the issues raised below are actually minor and/or matters of personal preference, several comments led us to improve and correct the figures and we thank this reviewer for the constructive suggestions.

(1) In Figure 1, it appears from the representative images that WIPI2 KD cells have higher levels of EGFR (Figure 1A and 1B). Is this correct?

To some degree. This increase is not systematic. A moderate increase has been observed only in 2 experiments out of 4. Therefore, we did not investigate this.

(2) Also in Figure 1, the colocalization is difficult to see. The authors should add the separate channels in addition to the merged images. Since the point is supposed to be that there is no impact on EGFR, all of this data could go into the supplement.

We had considered this already for the original version but dismissed the idea. The overlap is quantified in Fig. 1C, which provides the relevant values from four experiments. Fig. 1A/B provide only sample pictures, which also permit to see overlap (yellow) 0 and 5 min after the induction of degradation, which vanishes at later timepoints. Separating the channels would quadruple the space that this figure occupies, which would not be practical and not change the point to be made.

(3) The scale bars for each panel differ from each other. To better assess the data, the exact same magnification should be shown for each panel.

Corrected

(4) Figure 1C is confusing. The authors should explain which lines correspond to EEA1 and LAMP1.

Corrected

(5) In Figure 1D, the authors show different blots for control and WIPI2 KD. Could the authors compare WIPI2 and EGFR in the same blot? Without a comparison on the same blot, it is impossible to know whether the starting levels of EGFR are the same. Moreover, the quantitation in Figure 1E sets the value for each cell line to 100%. Instead, the starting levels in each cell line should be compared. The authors should use the amount of EGFR at zero time in the control cells to define 100%, and then indicate the relative initial EGFR levels in the WIPI2KD cells.

A new blot is shown now and the quantification has been performed as proposed.

(6) The quantification in Figure 1E does not match the representative blot shown in Figure 1D. According to the graph, the rate of degradation of EGFR is similar in both cell lines. But the representative blot shows that there are large differences.

We do not understand this comment. The representative blot shows similar kinetics for both. Perhaps the reviewer got confused by the fact that a marker lane was still present on the left blot and not labelled as such. The new version of the figure corrects this.

(7) The blot showing the WIP2 knockdown in Figure 1D has a lot of background. However, the blot of the WIPI2 knockdown in Figure S1 looks very good. The authors should make sure that they load enough sample and use a good antibody for the experiments in Figure 1.

The new blot that we added in response to comment 5 corrects this.

(8) In Figure 2 and Figure 3A, the cells are too confluent. This is an issue because the cells might not be metabolically active. In addition, the signal is saturated. The authors should make sure that all of the data is collected on cells that are not too confluent.

The confluency of the culture cannot be judged from single frames, which were selected to show several cells. We had controlled confluency and underlined in the Methods section that “For microscopy, the cells were plated on 18-mm-diameter glass coverslips on 24-well plates and grown for 2 or 3 days according to the protocol of DNA or siRNA transfection by reaching a confluency of 70-80%”. The reviewer may not have seen this.

(9) One main issue with these figures, especially the non-permeablized cells, is that it is impossible to assess how much of the signal is on the cell surface. The authors should provide the methods that they used to prevent inadvertent permeabilization of the cells. Were these experiments performed at 4 degrees? The authors should include a control of an antibody to a protein that is not found on the cell surface.

There is an internal control in that the non-permeabilised WIPI2KD cells, which have been treated with the same antibody, show no much less staining than the control cells (Fig. 3A). In WIPI2KD cells, integrin becomes accessible for antibody staining only upon detergent permeabilization. This demonstrates that our procedure does not lead to significant inadvertent permeabilization of the cells.

(10) The authors should perform surface biotinylation assays as an orthogonal approach to determine GLUT1 levels and beta1-integrin levels at the cell surface, respectively.

There is a strong, qualitative difference in the surface labelling of beta1-integrin that is not observed for GLUT1. Given that, it is not obvious to us what additional argument would be provided by surface biotinylation or subfractionation experiments.

(11) In quantifying surface levels of GLUT1 or beta1-integrin by microscopy, the authors should normalize to the cell area, rather than per cell.

The reviewer has probably not seen that the Methods section states that the cell area has been used for normalisation.

(12) In Figure 3, the nuclear DAPI stain in the KD cells is much less bright than in the control cells. The authors should make sure to choose representative images.

The nuclear DAPI signal has been visible in all cells. Depending on the position of the nucleus, is shape and dimension in the z-direction, individual nuclei can show different degrees of staining. The images shown are representative. We have adjusted the settings now to make the nuclei in the WIPI2KD cells easier to spot.

(13) For the immunofluorescence studies, the authors should be using single z planes rather than maximum projection.

Images have been exchanged by single planes.

(14) For the experiments in Figure 3, the authors should check the total levels of EEA1 and LAMP1 by western blot to test whether WIPI2 KD affects the levels of these proteins. If these organelle marker proteins are impacted, this could impact the colocalization measurements shown in Figures 3C and D.

We have measured the total fluorescence intensity of EEA1 and LAMP1 in the images. It shows no significant difference between control and WIPI2 knockdown cells (new Fig. 3F, H).

(15) In Figure 4A, the helical representation is rotated in the WIPI2-Sloop; the orientation of the residues that are not mutated should stay the same.

Yes. Done.

(16) In Figure 4B and 4C, cells that were not transfected with WIPI2 WT or WIPI2 Sloop should be shown.

Since the transfection efficiency is limited, the fields contain both non-transfected (lacking green fluorescence) and transfected cells (showing green fluorescence). We have now marked transfected cells with an asterisk.

(17) The cells in the lower panel of 4B have an unusual morphology and are much more round. The authors should choose cells that are representative of each experimental condition.

We now provide another field.

(18) In Figure 4C, it looks like the magnification of the top panels is different from the bottom panels. The same magnification for all the panels should be shown (and the size of the scale bars should be the same).

Corrected

(19) In Figure S4, the authors observe tubular structures. The authors should perform immunofluorescence with endosomal markers such as EEA1, LAMP1 and Retromer to determine the nature of the tubulovesicular structures.

We have done this (new Fig. S4). Rab4 is on tubules. Rab5 on the structures from which the tubules emanate.

(20) In Figure 5A, the top scale bar is missing.

Corrected.

(21) In Figure 5B, the confluency is too high.

See our response above. A single field does not permit to judge this. Confluency was controlled for all cultures. The cultures were not confluent.

(22) The IP studies shown in Figures 6, 7 and 8, should be accompanied by colocalization studies.

Colocalization measurments have now been integrated into the manuscript (Figs. S5, S6). They are consistent with the IP data.

(23) Figure 9 was very confusing and should be broken up into multiple figures. Data showing that localization did not change in any of the cell lines can be put in figures that are distinct from figures that show that localization changed in the various mutants. Figures that show no change can go in the supplement.

Since every panel of Fig. 9 shows a statistically significant difference we left the figure unchanged.

(23) Representative figures should be shown in the same figure as the corresponding graph. In addition, the order of the colocalization data shown in the graphs and figures should match the order described in the text.

We consider the graphs of Fig. 9 as the relevant information. Representative images are just illustration. Integrating them with the graphs would make it necessary to split everything up into multiple figures, making it harder to compare the different combinations. Therefore, we left the figures unchanged.

(24) In Figure S7, the Rab11 signal looks continuous, which makes the colocalization analysis meaningless. The authors should determine how to take images that can be evaluated. On a more minor note, the zoomed panels should be labeled as well.

This is a result of having shown a projections of multiple planes. The images have now been replaced by single plane images. Zoomed panels have been labelled and the scale bar added.

(25) The low colocalization of VPS35L with Rab5 is surprising, as SNX17 has been previously shown to co-localize with early endosomes positive for EEA1. This result may have occurred due to overexpression because the authors chose to utilize plasmids that express a tagged protein. There are antibodies to each of the endogenous proteins, and this is what should be used for this set of experiments.

This comment made us control the analysis performed for these images, which by mistake had been performed on z-projections rather than on single planes. This distorted the values. The re-analysed data shows a higher colocalisation with Rab5, but it remains inferior to colocalisation with Rab11.

(26) The authors should determine whether β1-integrin colocalizes with WIPI2 in endosomal compartments.

This was done. WIPI2 colocalizes with beta-integrin on EEA1-and SNX17-positive strcutures but not positive for LAMP1 (Fig. 3E/F).

Minor points

(27) In one of the panels in Figure 1A, "30 min" is duplicated.

Removed

(28) In Figures 5C and 5D, the y-axis should indicate that this is surface β1integrin.

Changed and added “surface”

(29) In Figure 9 there is a typo in panel A. It is VPS35L and not VPS35.

Corrected

**Reviewer #3 (Recommendations for the authors):**

This is an overall convincing study, which shows that the two complexes, CROP1 and CROP2 function at different membranes and serve different substrates. While I agree with their localization analysis, I have one key issue. The authors claim that each of the two forms a complex and base this on their specific pull-down and western blot analyses.

I find it important that they show that both indeed form stable complexes *in vivo*, using pull-down and mass spectrometry approaches. They have all the necessary tools in hand and could use WIPI1 and WIPI2 to demonstrate the existence of the two complexes. The FSSS mutants of each are good controls for such an analysis.

The manuscript actually presents the demanded *in vivo* experiments. Figs. 6 to 8 show pull-downs of WIPI1 and WIPI2 from cells, including also the FSSS mutant. While we haven't analysed this interaction by mass spectrometry, the Western blot analysis confirms the analysis. Cooperation of these proteins is further supported by the *in vivo* phenotypes, where the S67A substitution in WIPI2 produces a similar phenotype on integrin beta1 localisation as inactivation of Retriever.

A second aspect is the general presentation. The paper would be a lot more accessible if the subunits of each complex (CROP1 and CROP2) were also introduced in the figures of each part. For readers, a final model is helpful to put the data into context and show where each complex operates in the cell.

We have introduced a scheme of the respective complexes, including the names of the compunds, in Figs. 6 and 7 to avoid confusion.

Finally, it is not clear how the statistics compare to repeats in their data. This should be clarified.

This had been described in methods. Statistics has always been done on biological replicates stemming from independent experiments. We have added a cartoon (Fig. 10) depicting the trafficking pathways affected by CROP1 and CROP2.
